# Prevalence of sleep-disordered breathing and associations with orofacial symptoms among Saudi primary school children

**DOI:** 10.1186/s12903-019-0735-3

**Published:** 2019-03-12

**Authors:** Laila Baidas, Asma Al-Jobair, Huda Al-Kawari, Aram AlShehri, Sarah Al-Madani, Hana Al-Balbeesi

**Affiliations:** 1 Department of Pediatric Dentistry and Orthodontics, College of Dentistry, King Saud University, P.O. Box 5967, Riyadh, 11432 Saudi Arabia; 2 College of Dentistry, King Saud University, Riyadh, Saudi Arabia

**Keywords:** Sleep-disordered breathing, Obstructive sleep apnea, Pediatric sleep questionnaire, Asthma, Oral habits, Saudi children

## Abstract

**Background:**

This study aimed to determine the prevalence of sleep-disordered breathing among primary school children in Riyadh, Saudi Arabia, and to evaluate associations between sleep-disordered breathing and respiratory conditions/orofacial symptoms.

**Methods:**

In this cross-sectional study, 1600 questionnaires were distributed to Saudi boys and girls aged 6–12 years from 16 primary schools in Riyadh. The questionnaire covered relevant demographic and personal characteristics, presence of respiratory conditions and orofacial symptoms, and the Pediatric Sleep Questionnaire. The latter was used to assess the prevalence of symptoms of sleep-disordered breathing and was completed by the participating children’s parents.

**Results:**

In total, 1350 completed questionnaires were returned (85% response rate). The children’ mean age was 9.2 ± 1.8 years; 733 (54.3%) were boys and 617 (45.7%) girls. Overall, 21% of children were at high risk of sleep-disordered breathing. The prevalence of snoring was 14.4% and that of sleep apnea 3.4%. Boys were at higher risk of sleep-disordered breathing than girls (*P* = 0.040). Children with respiratory conditions or orofacial symptoms were at higher risk of sleep-disordered breathing (*P* < 0.0001) than children without these conditions/symptoms.

**Conclusions:**

Around 21% of Saudi children are at risk of sleep-disordered breathing. There is a strong association between sleep-disordered breathing symptoms and the presence of respiratory conditions or orofacial symptoms.

## Background

The term sleep-disordered breathing (SDB) covers a continuum of conditions ranging from primary snoring to severe obstructive sleep apnea (OSA) [1]. Sleep disturbances in children can lead to impairment of normal growth and to developmental problems that may affect behavioral and cognitive abilities [2]. Symptoms of SDB in children include snoring, difficulty breathing, mouth breathing, noisy breathing, sweating during sleep, and unusual sleeping positions that are adopted to overcome breathing difficulties. During the day, children with SDB are characterized by hyperactivity, restlessness, irritability, lack of attention, and loss of appetite [3, 4]. Risk factors for SDB in children are multidimensional and include sex, ethnicity, obesity, adenotonsillar hypertrophy, craniofacial abnormalities, exposure to cigarette smoke, respiratory infections, recurrent otitis media, allergic rhinitis, low socioeconomic status, and family history of snoring or OSA [5–9].

Habitual snoring, an important indicator of SDB in children, has been reported in primary school-aged children in various countries. In southern Italy, a study involving a large cohort of school- and preschool-aged children found that 4.9% of participants were habitual snorers and 1% had OSA [10]. A Turkish study reported a 7% prevalence of habitual snoring among children aged 6–13 years [11]. In addition, an 11.4% prevalence of habitual snoring was reported among Indian children aged 8–13 years [12] and a prevalence of 12% in Chinese schoolchildren [7]. A study involving Brazilian schoolchildren aged 9–14 years found that parents reported habitual snoring in 27.6% of children and apnea in 0.8% [13]. In Saudi Arabia, BaHammam et al. showed that sleep problems are prevalent among Saudi elementary school children, the prevalence of habitual snoring being 17% [14]. BaHammam et al. also investigated the prevalence of SDB among Saudi adults and found that 33% of middle-aged Saudi men and 39% of middle-aged Saudi women are at high risk of OSA [15, 16]. Al-Jewair et al. assessed the risk of OSA among Saudi adult dental patients and reported that 17% were habitual snorers and 23% at high risk of OSA [17]. Huynh et al. reported that 13.8% of a group of pediatric orthodontic patients were habitual snorers and 1.8% had sleep apnea [18].

The craniofacial development of children with SDB is similar to that of children with adenoid facies [19]. Any airway abnormality (e.g., hypertrophy of the adenoids) may lead to airway obstruction, chronic mouth breathing, and anatomical changes that affect the airway during active craniofacial development [20, 21]. These changes may increase airway instability and collapsibility, which can result in SDB and progression to OSA [22]. Children with SDB have distinct craniofacial morphology, including a posteriorly inclined mandibular plane angle, retrognathic mandible with increased lower anterior facial height, reduced posterior facial height, narrow maxillary arch with deeper palatal height, lateral cross-bites, and anterior open bite [18, 23, 24]. Therefore, it is important to consider any problems that contribute to poor skeletal and airway development in children, especially as these may have a negative impact on facial aesthetics and can be difficult to correct [25]. In addition to the orofacial characteristics associated with SDB, published reports suggest there is some association between SDB and orofacial symptoms. Oral habits such as digit sucking and bruxism, and facial pain such as temporomandibular disorder (TMD) are reportedly linked with SDB [26–28]. These relationships highlight the role of dentists in the early diagnosis, management, and referral of children with SDB [18, 29].

Increasing sleep problems among Saudi children highlight the need to investigate the prevalence of SDB in this population [14]. This study aimed to determine the prevalence of SDB symptoms in primary school children aged 6–12 years in Riyadh, Saudi Arabia, and to evaluate associations between SDB and respiratory conditions/orofacial symptoms.

## Methods

This cross-sectional study was conducted between September 2014 and May 2015 in Riyadh, Saudi Arabia. Approval was obtained from the Ethics Committee of the College of Dentistry Research Center at King Saud University (Registration No. IR 0105).

### Setting

Riyadh city is the capital of Saudi Arabia, which covers an area of 600 mile^2^ (1550 km^2^). The city has been subjected to high rates of population growth, from 150,000 inhabitants in the 1960s to over 5 million according to recent estimates [30].

### Calculation of sample size

A required sample size of 1300 was estimated based on a statistical power calculation of 85% and confidence level of 95%, assuming a SDB prevalence of 19% in Riyadh and 0.25 non-responses.

### Selection of schools

A list of all boys’ and girls’ public and private primary schools in Riyadh city was obtained from the Ministry of Education. These primary schools are responsible for providing education for children aged 6–12 years. A stratified randomization method was used to select schools for this study to ensure the sample was representative of schools in Riyadh. All public and private schools in every administrative district of Riyadh (North, East, West, South, and Central) were assigned a number. Schools were then selected randomly using a randomization Table. A total of 16 schools (10 public and six private) were selected: one public school for boys and one for girls from each district, and one private school for boys and one for girls from three districts (North, Central, and Eastern). These three districts were chosen because the majority of private schools are concentrated in these districts. The schools’ principals were invited to participate in this study via a letter explaining the purpose of the study and seeking their permission to distribute the questionnaires.

### Selection of children

In total, 1600 questionnaires were distributed to Saudi boys and girls aged 6–12 years. A similar sampling procedure to that used for school selection was conducted to select classes and students in each selected school. Children with known syndromes and compromised craniofacial anomalies were excluded from the study. Overall, 800 questionnaires were distributed to boys’ schools and 800 to girls’ schools.

### Questionnaire

The questionnaires were addressed to the children’s parents and sent home with each child. They included a cover page that explained the aim of the study and the importance of investigating the topic. Confidentiality of all information concerning the child was emphasized.

The questionnaire collected relevant demographic and personal characteristics (sex, age, school name, district, and parents’ educational level). The first part of the questionnaire included specific yes/no questions about the presence of specific respiratory conditions (e.g., asthma, respiratory allergy, tonsillitis, adenoiditis, and otitis media). This part of the questionnaire also assessed the presence of orofacial symptoms and included questions about digit sucking habits, tooth grinding during daytime and sleep, temporomandibular joint (TMJ) pain, and facial muscle pain. The second part of the questionnaire comprised the Pediatric Sleep Questionnaire (PSQ), which was used to assess the prevalence of symptoms of SDB. The PSQ includes 22 items that encompass prominent symptoms, such as snoring, breathing problems, mouth breathing, daytime sleepiness, and inattention/hyperactivity problems [4]. The original PSQ was translated into Arabic using the forward-backward translation method described by the World Health Organization [31].

A pilot test was conducted to ensure that the questions were correctly understood after translation. The translated questionnaires were first distributed to 10 mothers, who were asked to highlight any questions that were unclear. Some questions were adjusted in response to their feedback. A pilot study was then conducted with 50 parents of schoolchildren to test the reliability of the respiratory condition, orofacial symptom, and PSQ items. The parents involved in the pilot study were not included in the main sample. Item agreement was verified by the pilot study participants completing the questionnaire a second time after a 2-week interval. The kappa values were 0.87 for respiratory conditions, 0.89 for orofacial symptoms, and 0.91 for PSQ items.

Each PSQ item has three response options (yes, no, don’t know). Items are scored as one (yes) or zero (no or don’t know). On the basis of PSQ scores, children with 33% or more positive responses (scores of 8 or more) were considered at high risk of SDB, whereas children with fewer than 33% positive responses (scores less than 8) were considered at low risk of SDB.

### Data analysis

All statistical analyses were performed with SPSS version 22 (IBM SPSS Statistics for Windows, Version 22.0; IBM, Armonk, NY, USA). Participants’ characteristics and prevalence rates are reported using descriptive statistics. Bivariate analyses were performed using the χ^2^ test to assess differences between low- and high-risk children regarding age, sex, snoring, sleep apnea, mouth breathing, obesity, respiratory conditions, and orofacial symptoms. Univariate analysis and multivariate backward stepwise regression were performed to assess factors associated with the risk for SDB. *P*-values < 0.05 were considered to denote significance. The Hosmer–Lemeshow goodness-of-fit test and the area under the receiver operator characteristic (ROC) curve were used to assess the validity of the multivariate regression model.

## Results

In total, 1600 questionnaires were distributed to 16 primary schools in Riyadh city. Responses for 1350 children were received (85% response rate); 733 boys (54.3%) and 617 girls (45.7%). The children’s mean age was 9.2 ± 1.8 years. The children were divided into two age groups: 6–9 years (52.2%) and 10–12 years (47.8%). The highest response rate was from the Eastern area. The most common level of education for both parents was a bachelor’s degree. Mothers responded to 67.7% of the questionnaires.

In total, 21% of children were at high risk of SDB (8 or more “yes” responses on the PSQ). Habitual snoring (snoring all the time and always snoring during sleep) was reported in 14.4% of children, sleep apnea in 3.4%, mouth breathing in 21%, and overweight in 10.5% (Table 1).

**Table 1:** Distribution of participants with SDB symptoms (frequency and percentage)

Domain	Question	n (%)
Snoring/breathing problems	Snores all the time during sleep	50 (3.7)
	Always snores during sleep	145 (10.7)
	Snores loudly	151(11.2)
	Snores during day	113 (8.4)
	Difficulty in breathing	109 (8.1)
	Has stopped breathing during sleep	46 (3.4)
	Mouth breathing during day	284 (21.0)
	Dry mouth upon waking	289 (21.4)
	Wets bed, walks during sleep, or wakes up scared during night	274 (20.3)
Daytime sleepiness and development	Wakes up unrefreshed	403 (29.9)
	Wakes up with headache	130 (9.6)
	Difficult to wake child up	461 (34.1)
	Sleepiness during the day	329 (24.4)
	Sleepiness during the day noticed by teacher	133 (9.9)
	Has stopped growing at a normal rate	56 (4.1)
	Overweight	142 (10.5)
Inattention/hyperactivity	Does not respond quickly when spoken to	294 (21.8)
	Difficulty in organizing and managing tasks	276 (20.4)
	Easily distracted by external stimuli	589 (43.6)
	Seems restless and moves when seated	491 (36.4)
	Looks in a hurry all the time	541 (40.1)
	Interrupts others during speech	510 (37.8)
Number of children at high-risk of SDB (eight or more yes responses)		283 (21.0)

*SDB* sleep-disordered breathing

All sleep symptoms were strongly associated with high risk of SDB. Table 2 shows the characteristics that differed significantly between children at low- and high-risk of SDB. Boys were at higher risk of SDB than girls (*P* = 0.040). Habitual snoring, sleep apnea, mouth breathing, and being overweight were strongly associated with SDB (*P* < 0.001). All respiratory conditions and orofacial symptoms were more common in high-risk children (*P* < 0.001).

**Table 2:** Children’s characteristics according to risk of SDB

Variable			All children	Low-risk	High-risk	*P*-value
			*N* = 1350	*N* = 1067	*N* = 283	
			n (%)	n (%)	n (%)	
Personal characteristics	Sex	Female	617 (45.7)	503 (47.7)	114 (40.3)	0.040
		Male	733 (54.3)	564 (52.9)	169 (59.7)	
	Age group, years	6–9	705 (52.2)	555 (52)	150 (53)	0.767
		10–12	645 (47.8)	512 (48)	133 (47)	
SDB symptoms	Snoring	Yes	196 (14.4)	77 (39.3)	119 (60.7)	<0.001
	Sleep apnea	Yes	46 (3.4)	11 (1)	35 (76.1)	<0.001
	Mouth breathing	Yes	284 (21)	143 (13)	141 (49.6)	<0.001
	Overweight	Yes	142 (10.5)	78 (7.3)	64 (22.6)	<0.001
Respiratory conditions	Asthma	Yes	97 (7.2)	54 (5.1)	43 (15.2)	<0.001
	Respiratory allergy	Yes	214 (15.9)	139 (13)	75 (29.5)	<0.001
	Tonsillitis	Yes	186 (13.8)	119 (11.2)	67 (23.7)	<0.001
	Adenoiditis	Yes	108 (8.0)	48 (4.5)	60 (21.2)	<0.001
	Otitis media	Yes	102 (7.6)	54 (5.1)	48 (17)	<0.001
Orofacial symptoms	Digit sucking	Yes	114 (8.4)	70 (6.6)	44 (15.5)	<0.001
	Tooth grinding (day)	Yes	47 (3.5)	24 (2.2)	23 (8.1)	<0.001
	Tooth grinding (sleep)	Yes	126 (9.3)	78 (7.3)	48 (17)	<0.001
	Facial muscle pain	Yes	14 (1.0)	7 (0.7)	7 (2.5)	0.015
	TMJ pain on waking	Yes	31 (2.3)	7 (0.7)	24 (8.5)	<0.001

*SDB* sleep-disordered breathing, *TMJ* temporomandibular joint

Logistic regression analyses to evaluate possible risk factors for SDB are presented in Table 3. Children with habitual snoring, sleep apnea, or mouth breathing were at a four times higher risk of developing SDB. Overweight children were three times more likely to report SDB symptoms than other children (odds ratio [OR]: 3.3, 95% confidence interval [CI]: 2.1–5.2). Asthma was not significantly associated with SDB at the univariate level, but was significantly associated at the multivariate level. In contrast, tonsillitis was highly significantly associated at the univariate level but was not significantly associated at the multivariate level. Children who had adenoiditis were twice as likely to develop SDB as those without adenoiditis (OR: 2.4, 95% CI:1.0–2.9). Similarly, children with otitis media were at almost three times higher risk of developing SDB (OR: 2.6, 95% CI:1.5–4.5). Children who had digit sucking habits or sleep bruxism were at higher risk of SDB. Children with TMJ pain on waking were six times more likely to report SDB symptoms than other children (OR: 5.8, 95% CI: 2.1–16.3). All of these variables retained significance associations at the multivariate level. The Hosmer–Lemeshow test was not statistically significant (*P* = 0.135), and the area under the ROC curve was 0.840; both tests thus confirming the suitability of the tested model.

**Table 3:** Logistic regression analyses of SBD risk

Variables	Univariate analysis				Multivariate analysis			
	Odds ratio	95% Confidence interval		*P*-value	Odds ratio	95% Confidence interval		*P*-value
Snoring	4.319	2.864	6.513	<0.001	4.382	2.915	6.588	<0.001
Obstructive sleep apnea	4.406	1.888	10.281	0.001	4.280	1.833	9.996	0.001
Mouth breathing	4.388	3.104	6.203	<0.001	4.408	3.127	6.214	<0.001
Overweight	3.339	2.131	5.230	<0.001	3.326	2.122	5.212	<0.001
Asthma					1.798	1.058	3.054	0.030
Tonsillitis	1.770	1.058	2.971	0.030				
Adenoiditis	2.404	1.119	5.163	0.024	2.317	1.077	4.984	0.031
Otitis media	2.645	1.544	4.533	<0.001	2.607	1.537	4.424	<0.001
Digit sucking	2.768	1.680	4.559	<0.001	2.771	1.683	4.560	<0.001
Tooth grinding (day)	1.773	1.058	2.971	0.030	1.663	0.955	2.898	0.072
Tooth grinding (sleep)	1.958	1.185	3.234	0.009	1.998	1.214	3.289	0.007
TMJ pain on waking	7.250	2.331	22.546	<0.001	5.871	2.106	16.368	<0.001

*TMJ* temporomandibular joint

## Discussion

SDB affects children’s growth, development, cognitive function, and learning abilities, which may result in poor school performance [1, 2]. It is important to determine the prevalence of SDB in children because of its association with health problems such as hypertension, cardiovascular disease, and brain damage [32–34].

In the present study we used a validated questionnaire to assess the prevalence of SDB symptoms among Saudi primary school children aged 6–12 years in Riyadh city. The PSQ used in the present study was developed by Chervin et al., and is considered a valid and reliable instrument for identifying SDB risk, having a sensitivity of 81% and specificity of 87% [4].

The response rate was high (85%); only 10% of the questionnaires were not returned and 5% excluded because of incomplete information. The high response rate for the Eastern area of Riyadh city may be because it is more densely populated than the other included areas. The commonest level of parental education was a bachelor’s degree, possibly because educated parents are more aware of their children’s health problems and more willing to participate in research such as our study. Most questionnaires were responded to by mothers, which may be attributable to mothers being closer to their children and knowing more about their sleeping habits.

In this study, 21% of children were at high risk for SDB, 14.4% reported habitual snoring, and 3.4% reported sleep apnea. Sleep problems have previously been reported to be prevalent among elementary Saudi children, the reported prevalence of habitual snoring being 17% [14]. This rate is lower than that in a Dutch study, which reported that 25% of children aged 7–12 years had sleep problems [35], but is higher than rates reported in Italian (4.9%), Turkish (7%), and Indian (11.4%) schoolchildren [10–12]. However, it is lower than that reported in Brazilian schoolchildren (27.6%) [13]. In the present study, reported sleep apnea among Saudi schoolchildren was higher than that reported for Italian (1%) and Brazilian children (0.8%) [10, 13]. Mouth breathing was present in 21% of our sample, which is also higher than the 15.5% reported for Brazilian schoolchildren [13]. The varying reported prevalences of snoring, sleep apnea, and mouth breathing may be explained by differences in the targeted age groups, questionnaires used, definitions adopted, cohorts studied, and cultural factors.

Our study showed a male predilection regarding risk of SDB, with 23% of boys at high risk compared with 19% of girls. The findings of previous studies have been similar [7, 10, 11, 26]. However, one study found that girls were at a higher risk of experiencing sleep disturbances than boys [36] and several studies involving Brazilian and Indian schoolchildren did detected no differences between boys and girls in the prevalence of SDB symptoms [12, 13]. A systematic review by Lumeng and Chervin [1] concluded that the prevalence of childhood SDB differs according to sex, boys being more frequently affected than girls. Studies in which these differences were not clear may have had inadequate sample size and power to detect any difference. The large sample size of the present study allowed us to detect sex-based differences.

Many studies have investigated the association between respiratory conditions and SDB. We found that respiratory conditions (e.g., asthma, tonsillitis, adenoiditis, and otitis media) are common in children at high risk of SDB and the associations are strong. A previous study involving children aged 5–12 years in which breathing was monitored during sleep found that regardless of race, body mass index, and family history, symptoms of SDB were significantly associated with upper and lower respiratory symptoms [36]. Consistent with our study, Li et al. found a strong association between habitual snoring and several respiratory problems associated with atopy and infection, including chronic/allergic rhinitis, asthma, adenotonsillar hypertrophy, and otitis media [7]. Gozel et al. found an association between habitual snoring and high prevalence of recurrent otitis media among children aged 5–7 years [8]. The mechanisms underlying respiratory problems and habitual snoring are not yet clear. Respiratory problems may increase upper airway resistance and affect airway compliance, resulting in habitual snoring. In turn, habitual snoring may exacerbate some respiratory problems such as asthma by increasing cholinergic tone and promoting bronchial constriction [37, 38]. Repeated and frequent respiratory infections may also disturb a child’s normal sleep pattern and prevent normal nighttime endocrinological function and normal growth, as seen in children with sleep apnea [39, 40]. We found stronger associations between adenoiditis and otitis media and risk of SDB than between asthma and tonsillitis and risk of SDB. Given the prominent role of enlarged adenoids in increased upper airway resistance and in the pathophysiology of eustachian tube dysfunction [8], this difference may be attributable to the greater proliferation of lymphadenoid tissues in the upper airways in the former conditions.

Regarding oral habits, it is thought that non-nutritive sucking habits, (e.g., pacifier use and digit sucking) protects against SDB, possibly because they increase muscle tone and consequently decrease the collapsibility of the pharynx during sleep [41, 42]. However, in the present study we found an association between SDB and a history of digit sucking habits. This is consistent with the findings of Huynh et al., who reported that a statistically significant association between a history of thumb/finger sucking and heavy breathing at night [18]. In contrast, Al-Talib et al. evaluated the relationship between non-nutritive sucking habits and risk of SDB in a sample of 84 children aged 4–12 years and found that non-nutritive sucking habits had no effect on the prevalence of SDB [26]. Our study showed an association between SDB symptoms and tooth grinding. This contradicts the findings of Huynh et al., who reported that a habit of tooth grinding while awake or asleep is not associated with symptoms of SDB [18]. It has been suggested that tooth grinding or bruxism is a complex process and is associated with sleep position [27]. Associations between SDB, sleep position, and parafunctional activities (grinding, clenching) have been found in some patients [43]. A link between SDB and tooth grinding or bruxism is possible, given that these two conditions are both associated with alterations in muscle activity and tone [44]. Furthermore, facial pain reportedly occurs more frequently in children at high risk of SDB and children with TMJ pain are seven times more likely to have SDB than those who do not. Many studies have supported a significant association between symptoms of OSA and TMD [28, 45]. Although a causal association between TMD and SDB remains controversial, bruxism (which is more common in children at high risk of SDB than in those at lower risk) may be implicated in development of TMD [46]. The strong association between SDB and TMD suggests that children with TMD should be routinely screened for SDB and those with significant sleep problems strongly considered for referral for a complete sleep evaluation [45].

One limitation of our study is that the results are based on parents’ subjective views. Implementation of a more objective tool, such as polysomnography, would yield more accurate results. However, use of these tools may be impractical in large studies such as ours. Although our findings are comparable with those of previous studies, some contextual factors should be considered, including sample size, age group, type of questionnaire, variables tested, socioeconomic status, and ethnicity. Further studies that include children from different cities in Saudi Arabia are needed to determine whether our findings can be generalized to all Saudi children. Moreover, combining the use of questionnaires with clinical examinations of participating children may yield more information and thus take future studies to a higher level.

## Conclusions

We found that 21% of Saudi children are at risk of SDB, boys being at higher risk than girls. SDB symptoms (snoring, sleep apnea, mouth breathing, and being overweight), all investigated respiratory conditions, and orofacial symptoms were more common in high-risk children. Snoring, OSA, mouth breathing, overweight, otitis media, adenoiditis, digit sucking, tooth grinding, and TMJ pain on waking were all identified as risk factors. Thus, the significance of investigating SDB in younger age groups should not be overlooked.

## Abbreviations

OSA: obstructive sleep apnea
PSQ: Pediatric Sleep Questionnaire
SDB: sleep-disordered breathing
TMD: temporomandibular disorder
TMJ: temporomandibular joint
